# Global research trends on depression-related stigma in the 21st century: a bibliometric analysis

**DOI:** 10.1080/16549716.2025.2612390

**Published:** 2026-01-30

**Authors:** Lazzat Zhamaliyeva, Assemgul Batyrova, Nurgul Ablakimova, Galina Veklenko, Botakoz Malsova, Aidana Tautanova, Andrej M. Grjibovski

**Affiliations:** a Department of General Practice, West Kazakhstan Marat Ospanov Medical University, Aktobe, Kazakhstan; b Department of Propedeutics of Internal Disease, West Kazakhstan Marat Ospanov Medical University, Aktobe, Kazakhstan; c Department of Pharmacology, Clinical Pharmacology, West Kazakhstan Marat Ospanov Medical University, Aktobe, Kazakhstan; d Department of Microbiology and Virology, Astana Medical University, Astana, Kazakhstan; e Rectorate, Reaviz Universtiy, Saint Petersburg, Russia; f Department of Epidemiology and Modern Vaccination Technologies, I.M. Sechenov First Moscow State Medical University (Sechenov University), Moscow, Russia; g Department of Healthcare Organization and Preventive Medicine, M.K. Ammosov North-Eastern Federal University, Yakutsk, Russia; h Department of Health Policy and Management, Al-Farabi Kazakh National University, Almaty, Kazakhstan

**Keywords:** depression, stigma, mental health, bibliometric analysis, depressive disorder, social stigma

## Abstract

**Background:**

Depression is a leading contributor to the global burden of diseases. Stigma associated with mental illness significantly hinders help-seeking, diagnosis, treatment, and recovery. While research on mental health stigma has expanded over the past two decades, a systematic examination of its evolution, particularly in the context of depression, is almost non-existent.

**Objective:**

To map and analyze global research on depression stigma, focusing on publication trends, leading contributors, international collaborations, and thematic developments.

**Methods:**

We analyzed 947 peer-reviewed articles indexed in the Scopus database using bibliometric software in R-studio. Quantitative indicators included annual publication growth, citation analysis, leading countries, institutions, and authors, as well as international collaboration patterns. Additionally, keyword co-occurrence and thematic evolution analyses were conducted to explore conceptual developments within the field.

**Results:**

The number of publications steadily increased from 2013 to 2025. The United States, China, the UK, and Canada accounted for the highest research and citation impact, while contributions from low- and middle-income countries (LMIC) remained limited despite these regions carrying most of the global disease burden. Thematic mapping revealed a strong focus on clinical and psychosocial dimensions, with increasing attention to concepts such as resilience, social support, and the mental health effects of the COVID-19 pandemic in recent years.

**Conclusions:**

The volume of research on depression stigma has grown, yet significant geographical and conceptual disparities continue to persist. Strengthening collaboration, supporting LMIC research capacity, and integrating stigma reduction into global mental health frameworks are essential to achieving equitable mental health outcomes worldwide.

## Paper Context

- **Main findings:** Global research on depression stigma has increased steadily since 2013, with high-income countries leading output and LMICs countries remaining underrepresented.
- **Added knowledge:** This is the first bibliometric analysis to map 25 years of research on depression stigma, highlighting thematic evolution and collaboration networks.
- **Global health impact for policy and action:** Expanding inclusive and locally relevant research is essential for reducing disparities and informing culturally appropriate strategies to address depression stigma worldwide

## Background

Depression is one of the leading causes of disability worldwide, affecting mental health, daily functioning, and social well-being. The World Health Organization (WHO) ranks major depressive disorder as the third leading cause of global disease burden, projected to become the first by 2030 [1]. It is a major contributor to disability across low-, middle-, and high-income countries (LMICs) [2], impairing productivity, increasing suicide risk, and elevating the likelihood of chronic comorbidities [2,3]. The global magnitude of depression underscores the urgency of addressing stigma. More than 1 billion people globally live with mental health disorders such as depression and anxiety [4]. An important factor that exacerbates the burden of depression is mental health-related stigma. Already in 1963, Goffman described stigmatization as a process of discrediting, degrading, and devaluing. In essence, it involves the marginalization and diminished worth of an individual or group based on a distinguishing characteristic [5]. Stigma associated with depression can manifest in various forms of public stigma, self-stigma, and structural stigma and acts as a barrier to help-seeking, timely diagnosis, and effective treatment [6]. Individuals with depression often internalize societal prejudices and experience isolation, and hopelessness [7]. Stigmatizing attitudes among healthcare professionals can reduce empathy, lower expectations of recovery, and ultimately compromise the quality of care.

Despite the growing body of literature on depression and mental health stigma, the field remains fragmented. Bibliometric analysis provides a structured and objective approach to assessing research trends, identifying key authors, and mapping thematic developments. It allows to quantify research output, collaboration networks, and conceptual structures, thereby enabling researchers and policymakers to better understand the evolution and current state of research.

Although several systematic reviews have investigated interventions and determinants of stigma related to mental illness and depression among healthcare professionals and students [8–10], we failed to identify a comprehensive bibliometric analysis that captures the global research dynamics of this field. Previous scientometric studies have examined stigma within broader medical or social contexts; however, they have not specifically addressed depression-related stigma or its presence within primary health care research.

We conducted a bibliometric analysis of scientific publications on the stigmatization of mental health, especially depression. In particular, we studied the growth and trajectory of research results, identified the main journals, authors, institutions, and countries contributing to the field, and explored the thematic evolution and current research directions. This article is intended to serve as a basis for future research and interventions by identifying strengths, gaps, and opportunities within existing literature.

## Methods

### Data sources and search strategy

All bibliographic data were collected from the Scopus database. Scopus was chosen as the sole data source because it is one of the largest and most comprehensive multidisciplinary databases, providing extensive coverage of peer-reviewed journals in the health- and social sciences. It offers advanced search capabilities, high-quality indexing, and includes both global and regional journals relevant to mental health and primary health care. In addition, Scopus provides substantial overlap with other major databases, thereby increasing the likelihood of capturing a wide range of eligible studies [11]. The search strategy was developed to encompass various aspects of this field, including the conceptualization of depression, stigma, and their interrelation. Data collection was performed on 27 October 2025. The search strategy can be summarized as follows: (‘Depression’ OR ‘Major depressive disorder’) AND (‘Stigma’ OR ‘Shame’ OR ‘Disgrace’ OR ‘Discrimination’). The search was applied to the title field (see Table 1). The time frame was restricted to 2000–2025, with no limitations on country, language, or study population, in accordance with Scopus indexing practices. For this review, mental health was considered in a broad sense, with depression selected as the primary focus due to its high global prevalence and central role in mental health stigma research. A flow diagram of the literature search and screening process was created to illustrate the stages of identification, screening, eligibility assessment, and final inclusion.

**Table 1.** Search strategy in Scopus.

No	Queries	Search result
1	‘Depression’ OR ‘Major depressive disorder’ (Title)	213,521
2	‘Stigma’ OR ‘Shame’ OR ‘Disgrace’ OR ‘Discrimination’ (Title)	103,556
3	#1 AND #2	1073

### Drawing figures of the manuscript

Bibliographic data were retrieved from Scopus on 27 October 2025. Duplicate records were identified and removed prior to analysis. To ensure the reliability of data extraction and processing, two researchers independently verified the dataset and cross-checked results for consistency.

Bibliometric analysis and science mapping were conducted using the *Bibliometrix* package in RStudio [12].

### Data extraction and analysis

All records were ranked according to total citation counts. The ten most cited papers were identified and examined in detail to determine their primary research themes, target populations, and contributions to advancing knowledge.

### Annual scientific production/number of publications

Annual publication output and citation patterns were analyzed to assess temporal trends in research activity. The number of publications per year was plotted to identify changes in scholarly productivity, while average annual citations per article were calculated to evaluate citation dynamics over time.

### Core journals (Bradford’s law)

Bradford’s law was applied to identify the ‘core’ journals in the field [13]. Journals were ranked in descending order according to the number of relevant publications. The ranked list was divided into Bradford zones, each containing a similar cumulative proportion of total articles. The Bradford core (zone 1) was defined as the smallest set of journals contributing the highest number of publications. Visualization was performed using a Bradford plot, where the cumulative number of publications is plotted against the journal rank, with the core zone shaded.

### Leading institutions, sources, authors, and collaborating countries

The ten most productive institutions and authors were identified and ranked according to their share of total publications. Collaboration patterns between institutions and authors were examined through network visualizations. For the country-level analysis, nations were ranked based on their proportion of total articles, and the multiple-country publication (MCP) rate was calculated for the top ten countries to assess the extent of international collaboration. The international collaboration network was visualized using co-authorship data between countries, highlighting the strength and direction of global research partnerships.

### Keyword co-occurrence

Keyword co-occurrence analysis was based on author-supplied keywords. Keywords were standardized to lowercase, and synonymous terms were merged. A co-occurrence network was generated using a minimum frequency threshold of 10, and clustering was performed using the Louvain algorithm within the Bibliometrix framework to identify major thematic clusters.

### Thematic evolution analysis

To examine the temporal evolution of research topics, a thematic evolution map was generated using the Bibliometrix package in RStudio with its Biblioshiny interface. The analysis was based on author keywords, segmented into time slices to identify emerging, declining, and persistent themes. Links between themes were mapped to visualize conceptual transitions over the study period.

## Results

### Study selection

**Figure 1.**
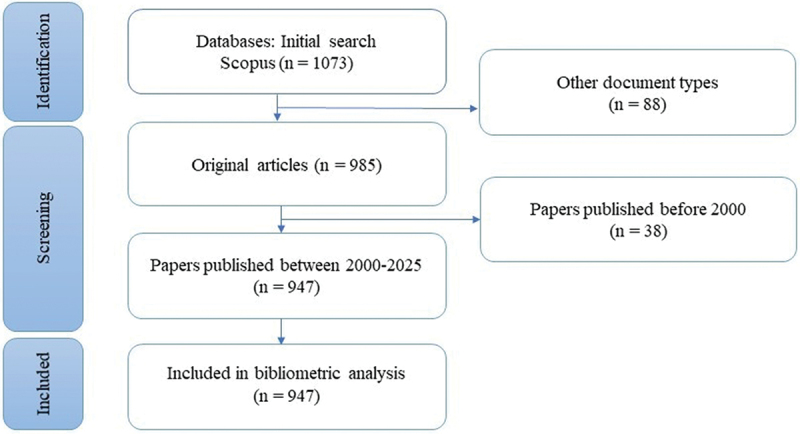
Flow diagram of the literature search and screening process.

The database search initially yielded 1073 records. After removing publications that did not meet the inclusion criteria, a total of 947 articles remained eligible for the bibliometric analysis. The selection process is illustrated in Figure 1.

### Overview of the papers

This study covered the period 2000–2025 and included 947 journal articles published across 480 academic journals. In total, 4127 authors contributed to these publications, with an average of 33.27 citations per article. These findings highlight sustained scholarly interest in the psychosocial and clinical dimensions of mental health stigma. The citation impact analysis identified the ten most cited papers (Table 2), which collectively shaped research priorities by addressing key themes such as the influence of stigma on help-seeking behavior, treatment adherence, suicidal ideation, and its impact on vulnerable populations.

**Table 2.** The 10 most globally cited articles in the field of mental health stigmatization (2000–2025).

Authors	Title	Journal	Citations	DOI
Barney et al. [14]	Stigma about Depression and its Impact on Help-Seeking Intentions	*Australian & New Zealand Journal of Psychiatry*	630	10.1111/j.1440–1614.2006.0174.x
Sirey et al. [15]	Perceived Stigma as a Predictor of Treatment Discontinuation in Young and Older Outpatients With Depression	*American Journal of Psychiatry*	587	10.1176/appi.ajp.158.3.47916
Finch et al. [16]	Perceived discrimination and depression among Mexican-origin adults in California	*Journal of Health and Social Behavior*	582	10.2307/2676322
Gilbert [17]	The relationship of shame, social anxiety and depression: the role of the evaluation of social rank	*Clinical Psychology & Psychotherapy*	540	10.1002/1099–0879(200007)7:3 < 174:AID-CPP236 > 3.0.CO;2-U
Simbayi et al. [18]	Internalized stigma, discrimination, and depression among men and women living with HIV/AIDS in Cape Town, South Africa	*Social Science & Medicine*	487	10.1016/j.socscimed.2007.01.006
Schwenk et al. [19]	Depression, Stigma, and Suicidal Ideation in Medical Students	*JAMA*	443	10.1001/jama.2010.1300
Conner et al. [20]	Mental Health Treatment Seeking Among Older Adults With Depression: The Impact of Stigma and Race	*American journal of Geriatric Psychiatry*	431	10.1097/JGP.0b013e3181cc0366
Schulz et al. [21]	Discrimination, symptoms of depression, and self-rated health among african american women in detroit: results from a longitudinal analysis	*Public Health*	371	10.2105/AJPH.2005.064543
Lasalvia et al. [22]	Global pattern of experienced and anticipated discrimination reported by people with major depressive disorder: a cross-sectional survey	*Lancet*	346	10.1016/S0140-6736(12)61379–8
Griffiths et al. [23]	Predictors of depression stigma	*BMC Psychiatry*	338	10.1186/1471-244X-8–25

Across these influential studies, a consistent focus was placed on the psychosocial mechanisms and behavioral consequences of stigma related to depression. Notably, perceived and internalized stigma were found to negatively affect individuals’ willingness to seek help, maintain treatment, and achieve recovery.

Several highly cited studies further examined how shame, self-evaluation, and social comparison processes contribute to the persistence of depressive symptoms and avoidance behaviors. Another recurring theme concerns the intersectionality of stigma, particularly how discrimination is related to race, age, or chronic illness – including Human Immunodeficiency Virus/Acquired Immunodeficiency Syndrome (HIV/AIDS), compounds depressive experiences and social marginalization. Taken together, these findings emphasize that depression-related stigma is a multidimensional phenomenon, shaped by psychological, social, and structural factors, highlighting the need for context-specific and population-tailored interventions.

### Trend of publication and citation

**Figure 2.**
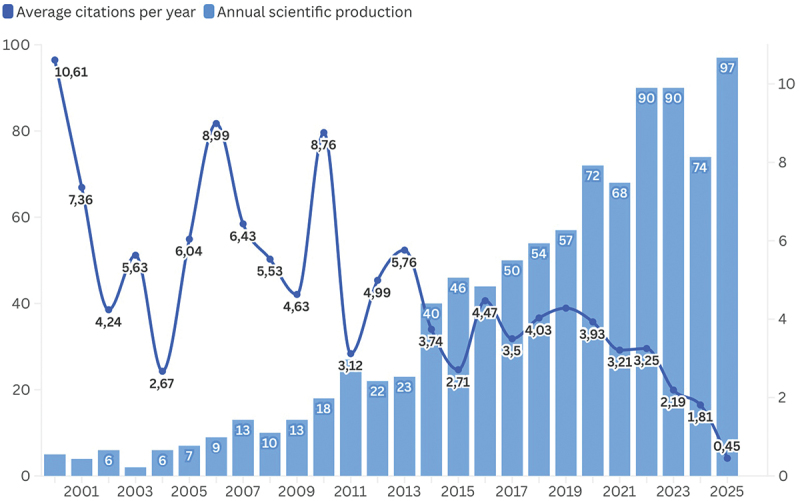
Annual trends in the number of publications and the average number of citations per year in the field of mental health stigmatization (2000–2025).

Annual trends in publication output and citation frequency on depression and mental health stigma between 2000 and 2025 indicate a steady rise in research activity (Figure 2). The number of publications increased gradually from the early 2000s, with marked acceleration after 2013 and a peak of 97 papers in 2025. In contrast, the average annual citation rate fluctuated considerably, reaching its highest levels in 2001, 2007, and 2010, followed by a gradual decline from 2015 onward. The sharp decrease in recent years, dropping to 0.45 in 2025, likely reflects the citation lag associated with newly published articles and the diversification of the research field.

**Figure 3.**
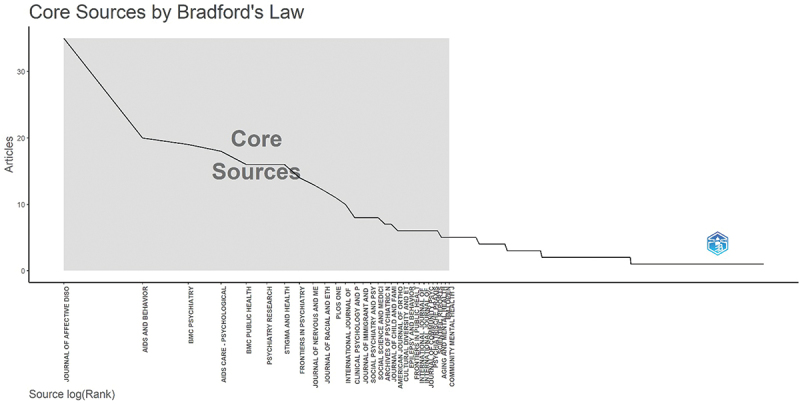
The plot of Bradford’s law identified core journals on mental health stigmatization, highlighting the most productive sources in the field (2000–2025).

The application of Bradford’s law identified a total of 26 journals contributing to the literature on depression-related stigma. The core zone comprised 10 journals – Journal of Affective Disorders, AIDS and Behavior, BMC Psychiatry, AIDS Care – Psychological – which together published 179 articles (32.08%). The complete list of the most productive sources is presented in Figure 3.

### Most productive authors, institutions, countries and their collaboration network

**Figure 4.**
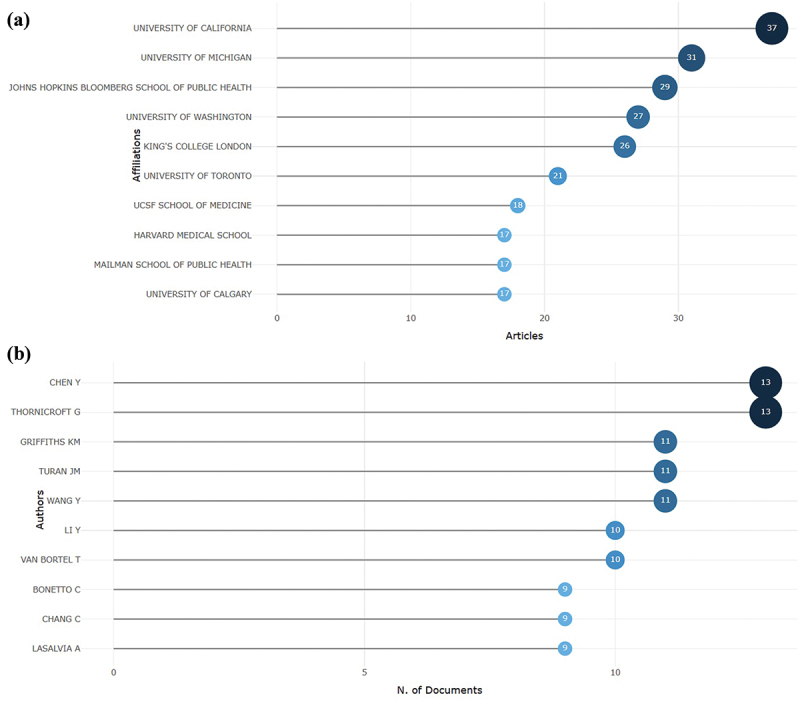
(a) Leading institutions, authors, countries, and their collaborative network. (b) Top ten contributing authors and their publication output in 2000–2025.

The institutional structure of research in this field demonstrates a clear dominance of well-established academic centers in North America and Europe. The University of California leads with the highest number of publications (37), reflecting its long-standing engagement in mental health and behavioral science research. Close behind are the University of Michigan (31) and the Johns Hopkins Bloomberg School of Public Health (29), both recognized for their interdisciplinary work bridging psychiatry, psychology, and social determinants of health. King’s College London (26) and the University of Toronto (21) further underscore the strong involvement of UK and Canadian institutions in advancing global mental health scholarship. Although the University of Washington contributed fewer publications (7), its work frequently appears in high-impact journals, highlighting an emphasis on research quality over quantity. Collectively, these universities form the intellectual core of the field, driving collaborative networks and shaping international research trajectories (Figure 4(a)).

At the author level, productivity is concentrated among a small core group of highly collaborative researchers, suggesting the existence of well-established research networks that define the field’s agenda. To assess relative impact, the H-index was applied, identifying authors with the most significant and sustained scholarly contributions. Chen Y and Graham Thornicroft demonstrated the highest H-index (13), followed by Kathleen M. Griffiths, Jennifer M. Turan, and Wang Y (11). Other notable contributors, including Li Y, Van Bortel, Bonetto C, Chang C, and Lasalvia A, also showed strong and consistent scholarly influence with H-indices around 9. This concentration of intellectual leadership indicates that the field is guided by a limited number of highly cited scholars, whose collaborative networks play a pivotal role in advancing global research on depression and mental health stigma (Figure 4(b)).

**Figure 5.**
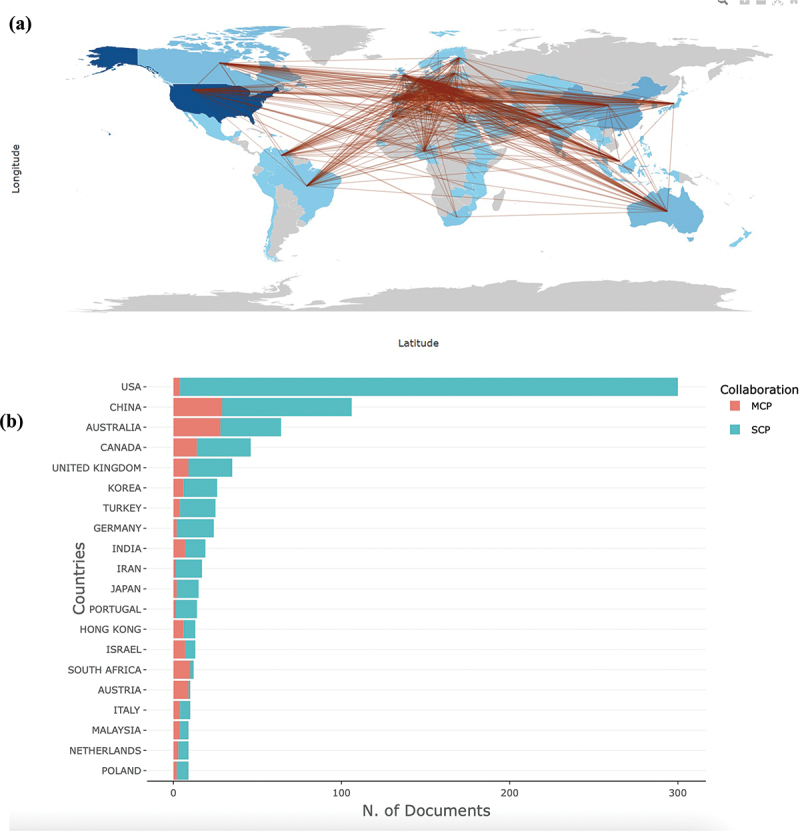
(a) Global collaboration in mental health stigma research, 2000–2025. Darker shading indicates countries with higher publication output, and thicker connecting lines represent stronger collaboration ties. (b) Top contributing countries in research on depression and mental health stigma (2000–2025), classified by single-country (SCP) and multi-country (MCP) collaboration.

The international collaboration network revealed a dense and multidirectional pattern of scientific partnerships (Figure 5(a)). The United States was the most productive and central contributor, producing 1317 publications and maintaining collaborative ties with over 70 countries, including China, the United Kingdom, Canada, Australia, and Germany (Table 3). China was second (361) and demonstrated extensive bilateral collaborations with both Western and Asian partners, particularly the United States, the United Kingdom, and India. Other highly productive countries – Canada (150), the United Kingdom (148), Australia (124), Germany (93), Italy (93), Turkey (81), India (63), and the Netherlands (58) – also formed strong regional and transcontinental links, often serving as bridges between high- and middle-income research settings.

**Table 3.** Leading publishing countries on mental health stigmatization (2000–2025).

Country	Number of Articles
USA	1317
China	361
Canada	150
United Kingdom	148
Australia	124
Germany	93
Italy	93
Turkey	81
India	63
Netherlands	58

The co-authorship map illustrates that research activity is dominated by North American and European clusters, with the USA-UK-Canada-Australia-China axis representing the most active collaboration hub. European countries such as Germany, Italy, and the Netherlands play a key role in connecting Central and Eastern Europe, while India, Turkey, and Malaysia represent emerging nodes facilitating South-South and cross-regional collaborations. Despite a growing number of contributors from Africa, the Middle East, and South Asia, their collaborations remain largely mediated through partnerships with high-income countries. This pattern is further supported by the country productivity and collaboration analysis (Figure 5(b)), which shows that the United States and China not only lead in total research output but also in the proportion of multi-country publications (MCP), reflecting their central role in fostering global collaboration. In contrast, many other contributing nations, particularly those in Asia and the Global South, exhibit predominantly single-country publications (SCP), suggesting limited integration into broader international research networks. Overall, the network reflects a globalized yet asymmetrical collaboration structure, where high-income countries serve as central hubs driving research productivity and international cooperation, while LMICs participate mainly through cross-border partnerships rather than independent production.

**Figure 6.**
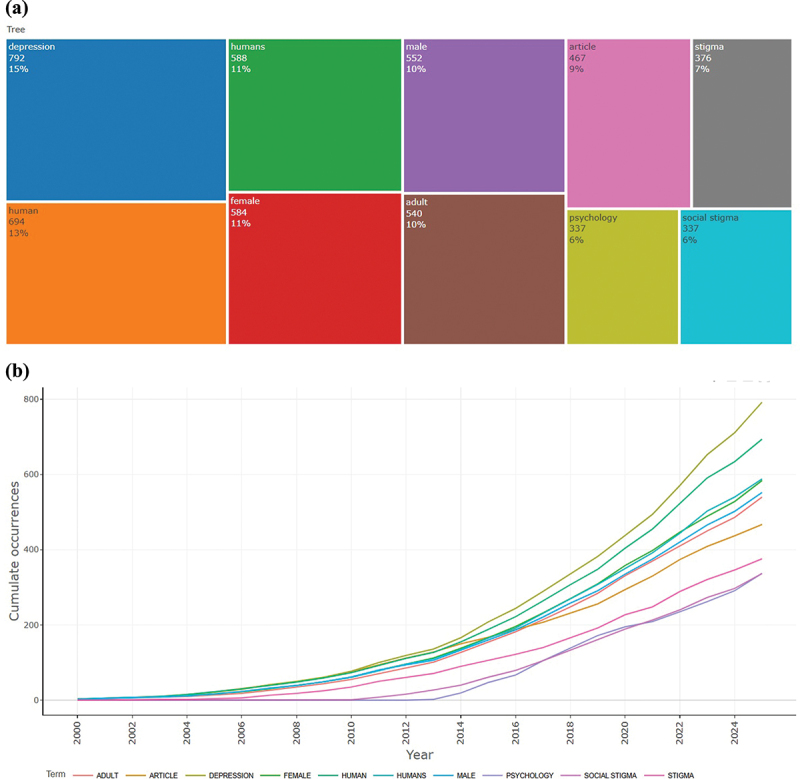
TreeMap (a) and scatter plot (b) representing the top ten author keywords in research on depression and mental health stigma (2000–2025).

The temporal analysis of author keywords (Figure 6(a,b)) reveals that the field is conceptually anchored in terms directly related to the disorders, demographic descriptors, and psychosocial constructs. Alongside core terms such as depression, stigma, and social stigma, a substantial proportion of keywords refer to demographic categories, including female, male, humans, and adult. The prominence of these descriptors indicates that much of the existing research adopts a population-based focus, with considerable attention given to gender differences and age-specific issues, particularly in older adults.

Longitudinal trends show a marked expansion in keyword diversity after 2010, with a particularly sharp increase in the past decade. This pattern reflects an increased interest in stigma-related mental health challenges and a gradual broadening of thematic coverage to encompass social, demographic, and psychological perspectives.

**Figure 7.**
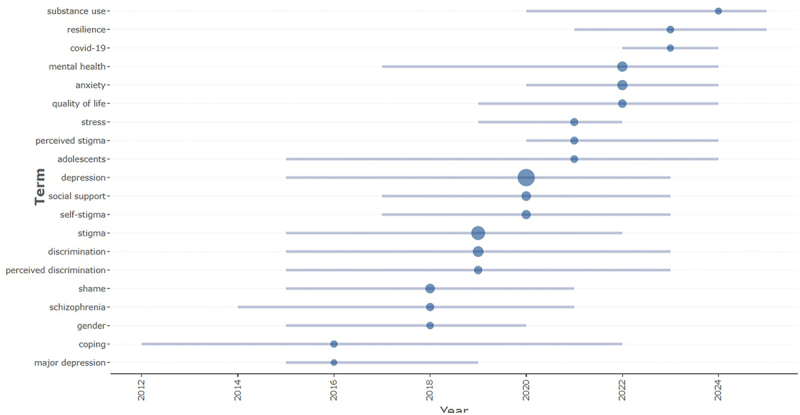
The timeline of the trend topics. Each bubble indicates the peak of frequency used for each, while the line indicates the years it was used.

The timeline analysis (Figure 7) shows the evolution of key terms in depression and mental health stigma research from 2012 to 2025. Early studies focused on clinical aspects such as major depression, coping, and schizophrenia, while later years emphasized stigma, social support, and self-stigma. Recent publications increasingly highlight resilience, quality of life, anxiety, and СOVID-19, reflecting a broader psychosocial and contextual focus.

## Discussion

Our study provided a comprehensive overview of global research on the stigmatization of mental health, particularly depression, revealing both thematic strengths and structural inequities in the field. Annual bibliometric analyses are increasingly valued for their ability to track intellectual progress and thematic evolution within specific research domains [24]. Moreover, they help to identify emerging priorities and guide translational applications in clinical and public health contexts [25].

The most influential studies consistently document the harmful impact of stigma on help-seeking and treatment adherence. Individuals with depression often delay or avoid care due to fear of judgment, shame, or discrimination. Among medical students [26], older adults, and people living with HIV, perceived and internalized stigma lower self-esteem, fosters social isolation, and aggravates depressive symptoms, posing major obstacles to recovery [27,28].

Another recurring topic is the intersection between depression, chronic conditions such as HIV/AIDS, and sociodemographic factors like age, gender, and race to deepen marginalization. Studies from South Africa [29–31] and Thailand [32] illustrate how overlapping stigmas amplify both the risk and the impact of depression. Highly cited works highlight older adults and medical students as particularly vulnerable populations, underscoring the need for tailored anti-stigma strategies suited to their psychosocial realities [19,20,28].

Keyword co-occurrence analysis confirms the field’s strong psychosocial orientation, with central terms such as depression, stigma, social stigma, and psychology. However, there remains a notable gap – few influential studies explicitly link these constructs to community-based health approaches and anti-stigma interventions. For instance, a recent scoping review [33] showed that although community-based psychosocial support interventions hold promise, few have been rigorously evaluated for stigma reduction [34]. Demographic descriptors such as *male*, *female*, and *adult* continued attention to population-specific vulnerabilities, particularly among adults and older adults. This focus aligns with global demographic shifts and aging-related mental health needs. However, children, adolescents, displaced persons, and indigenous communities remain largely overlooked, even though early-life adversity, migration, and cultural marginalization strongly influence mental health trajectories [35]. Further research should include these groups and adopt more community-based, participatory approaches to make future anti-stigma efforts more inclusive and globally representative.

The institutional landscape shows a strong concentration of output within well-established academic centers in North America and Western Europe. Leading institutions such as the University of California, the University of Michigan, and Johns Hopkins Bloomberg School of Public Health consistently rank among the most productive contributors. Similar patterns have been observed in other bibliometric studies, where Anglo-American universities dominate global mental health research due to their greater access to funding, international collaboration networks, and established publication infrastructure [36,37]. At the author level, the field is shaped by a small core of highly collaborative and influential scholars, including Chen Y [38,39] and Graham Thornicroft (H = 13) [27], Kathleen M. Griffiths, Jennifer M. Turan, and Wang Y. (H = 11), whose sustained contributions have defined much of the field’s direction [14,39–45].

LMICs remain underrepresented in the literature, even though they account for more than 80% of the world’s population affected by mental disorders [46]. According to the WHO (2020), mental health expenditures in LMICs represent only 1%–1.6% of total healthcare spending [47,48]. These structural inequities restrict access to care and shape the geography of scientific visibility, reinforcing North-South disparities in both knowledge production and policy influence.

The thematic evolution analysis supports this imbalance, showing that concepts particularly relevant to resource-limited settings – such as resilience, social support, and community engagement – have gained prominence only in recent years. While this trend is encouraging, the practical integration of these psychosocial factors into community health and anti-stigma interventions remains limited. Strengthening these links could enhance the applied value of stigma research, especially where health systems are under-resourced. Journals and funding agencies can play a key role by promoting equitable publication practices, supporting open-access initiatives, and prioritizing cross-regional collaborations. Researchers, in turn, should pursue participatory, community-based methods that translate conceptual insights into actionable stigma-reduction strategies. These findings also align with the WHO Mental Health Action Plan (2013–2030) [49], which calls for equity, community-based approaches, and strengthened research capacity in low-resource contexts. Linking bibliometric evidence to such frameworks can guide funders and policymakers in supporting equitable, sustainable mental health research systems. Strengthening LMIC research infrastructure, promoting South-South and North-South collaborations, and integrating stigma-reduction into primary care align with the WHO’s goals for universal access and parity in mental health services.

Overall, this bibliometric analysis underscores the urgent need for inclusive and contextually grounded research on depression-related stigma. Expanding global collaborations and diversifying methodological and geographic representation are essential for a fairer distribution of knowledge. Embedding stigma research within broader global health agendas can foster progress toward more equitable and culturally responsive mental health systems.

### Advantages and limitations

This study presents several important strengths. It is the first bibliometric analysis to focus specifically on the intersection of depression and mental health stigma, offering a targeted synthesis of a rapidly growing research field. Second, it uses a robust methodological approach by applying the Bibliometrix package in RStudio, enabling advanced mapping of bibliographic data, thematic evolution, and collaboration networks. Finally, it spans a 25-year period (2000–2025), capturing both historical trends and emerging themes, including the impact of the COVID-19 pandemic on research output.

However, the study relies exclusively on the Scopus database, which may not include relevant publications indexed in other databases. While bibliometric tools provide valuable quantitative insights, they cannot fully capture the qualitative depth and sociocultural complexity of stigma-related research. The reliance on keyword frequency and citation counts may also underrepresent innovative or recently published studies that have not yet accumulated significant academic attention. Furthermore, some articles included in this analysis were indexed in Scopus as ‘in press’ at the time of data extraction, and their citation counts may not yet accurately reflect their scientific impact.

Despite these limitations, the study provides a foundation for future research and policy planning. It highlights critical gaps in the literature and calls for more inclusive, interdisciplinary, and context-sensitive approaches to address stigma and mental health disparities worldwide.

## Conclusions

This bibliometric review provides a comprehensive overview of global research trends on depression and mental health stigma over the past 25 years. Research remains concentrated in high-income countries, underscoring the need to strengthen collaboration and research capacity in underrepresented regions. Aligning future studies with global mental health frameworks and promoting equitable, community-based approaches will be essential to reduce stigma and improve mental health outcomes worldwide.

## Data Availability

The datasets generated and analyzed during the current study are available from the corresponding author on reasonable request.
